# Low-Temperature Magnetocaloric Properties of V12 Polyoxovanadate Molecular Magnet: A Theoretical Study

**DOI:** 10.3390/ma13194399

**Published:** 2020-10-02

**Authors:** Karol Szałowski

**Affiliations:** Department of Solid State Physics, Faculty of Physics and Applied Informatics, University of Łódź, ulica Pomorska 149/153, PL90-236 Łódź, Poland; karol.szalowski@uni.lodz.pl

**Keywords:** magnetocaloric effect, magnetic entropy, molecular magnet, magnetic cluster, isotermal entropy change, magnetic Grüneisen ratio, tetramer, polyoxovanadate

## Abstract

The paper presents a computational study of the magnetocaloric properties of the V12 polyoxovanadate molecular magnet. The description is restricted to low-temperature range (below approximately 100 K), where the magnetic properties of the system in question can be sufficiently modelled by considering a tetramer that consists of four vanadium ions with spins S=1/2. The discussion is focused on the magnetocaloric effect in the cryogenic range. The exact and numerical diagonalization of the corresponding Hamiltonian is used in order to construct the thermodynamic description within a version of the canonical ensemble. The thermodynamic quantities of interest, such as magnetic entropy, specific heat, entropy change under isothermal magnetization/demagnetization, temperature change under adiabatic magnetization/demagnetization, refrigerant capacity, and magnetic Grüneisen ratio, are calculated and discussed extensively. The importance of two quantum level crossings for the described properties is emphasized. The significant ranges of direct and inverse magnetocaloric effect are predicted. In particular, the maximized inverse magnetocaloric response is found for cryogenic temperatures.

## 1. Introduction

Molecular magnetism [1,2,3], being an emerging and rapidly developing field, attracts the concerted efforts of physicists and chemists that aim at the design and synthesis of novel materials and mastering the methods of their theoretical and experimental characterization. Among a huge variety of structures, polyoxometalates are known to provide a flexible platform for obtaining molecular magnetic clusters of diverse properties [4,5,6,7]. Within this group, polyoxovanadates constitute an extraordinarily interesting class of materials [8,9]. This group of highly symmetric, weakly interacting cluster-based structures includes such frequently investigated members as V15 [10], V6 [11], or V3 [12] clusters. Additionally, an exhaustively studied archetypical group of V18 nearly spherical clusters with tunable electron population without structural changes can be mentioned [9].

A highly interesting structure from the above-mentioned group is the molecular magnet abbreviated as V12. It belongs to a class of mixed valence polyoxovanadates [13] with geometry that is based on a spherically shaped cluster. The abbreviation V12 collectively names three similar chemical compounds, the magnetic core of which is based on 12 vanadium ions with valence V${}_{8}^{\mathrm{IV}}$V${}_{4}^{V}$[13] included in [V${}_{12}$As${}_{8}$O${}_{40}$(H${}_{2}$O)]${}^{4-}$ ion. All of these three compounds share the structure in which V ions form three stacked tetramers and the inner tetramer with 4 V ions carrying spins S=1/2 originating from *d* electrons [14] is responsible for the low-temperature magnetic properties [13]. The most frequently studied compound of V12 type is (NH(C${}_{2}$H${}_{5}$)${}_{3}$)${}_{4}$[V${}_{8}^{\mathrm{IV}}$V${}_{4}^{V}$As${}_{8}$O${}_{40}$(H${}_{2}$O)]·H${}_{2}$O, which we will focus our attention on in the further discussion.

Magnetic tetramers composed of spins S=1/2 in all possible geometries—linear chain, rectangular planar structure, or tetrahedron—have attracted so far noticeable efforts of theorists. In this context, the studies focused on magnetic susceptibility [15], neutron scattering properties [15,16] correlations [17], and quantum entanglement [16,18,19,20] can be mentioned. In addition to these calculations, a material-oriented, Density Functional Theory (DFT) calculations aimed at prediction of energy levels of V12 were performed [14]. Also a theoretical and experimental study of Ni-based tetramer can be found [21].

The V12 polyoxovanadate molecular magnet has been studied experimentally with a range of methods in order to achieve relatively complete physical picture. Such approaches as the studies by neutron scattering [13,22], nuclear magnetic resonance aimed at characterization of spin-lattice relaxation [23,24,25,26], studies of magnetic susceptibility [13,23], or studies of transport and optical properties [14] can be mentioned in this context.

Among a plethora of prospective applications and functionalities [27], molecular magnets can serve as highly promising magnetocaloric materials [28,29] that exhibit noticeable magnetocaloric effect (MCE). This phenomenon, which is based on the entropy dependence of the external magnetic field, is of significant interest due to its applications in modern solid-state cooling [30]. Within this context, molecular magnets offer a possibility of designing the magnetocaloric properties [31] and exhibit a degree of multifunctionality [32]. The optimization of magnetocaloric performance of molecular magnets [33,34,35] involves, for example, strategies based on exploiting the frustration [36,37], achieving particularly high spins [38] or utilizing magnetic anisotropy in rotational MCE [39]. One of the approaches can be connected with the pronounced sensitivity of the magnetic entropy to the magnetic field variation in the vicinity of the quantum level crossing [40] in magnetic clusters [41]. This can serve as a motivation for the exploration of the thermodynamic properties of spin clusters with various geometries from the magnetocaloric point of view, especially with the use of the exact methods.

The theoretical works concerning the exact characterization of magnetocaloric properties of the model magnetic clusters that are composed of spins S=1/2 involve, for example, various Ising systems, to mention regular polyhedra [42,43], tetrahedra [44] or triangle-based clusters [45,46,47]. In addition, the Heisenberg clusters were also studied, such as regular polyhedra [48], cuboctahedron [49], or cupolae [50]. In that context, we can also mention our recent works concerning particular molecular magnets, such as V6 polyoxovanadate [51] or Cu-based hourglass-shaped cluster [52], both based on triangular units.

One of the temperature ranges of fundamental interest for MCE applications is a cryogenic range appropriate for operation of modern elaborate low-temperature nanodevices, to mention even the extreme applications for on-chip cooling [53,54,55]. The molecular magnetic materials are constantly mastered for the application in this range and they belong to the most promising systems applicable for efficient cryogenic cooling [31,34,56]. Therefore, a quest for novel materials offering good performance in this field and the prediction of the relevant properties of already known molecular materials is desirable.

Motivated by this fact and willing to supplement the physical picture of V12 polyoxovanadate properties, here we present a computational study of the magnetocaloric properties of this molecular magnet. We restrict our interest to the low-temperature range (below 100 K), where the magnetic properties of the system in question can be sufficiently modelled by considering a central tetramer. Particular attention is focused on the range of lowest temperatures, below 10 K, with a view to predict the applicability of V12 for cryogenic cooling. We use exact analytic and numerical diagonalization of the corresponding Hamiltonian to construct the thermodynamic description within a version of the canonical ensemble, as explained in detail in the Section 2. The thermodynamic quantities of interest from the MCE point of view, such as magnetic entropy, specific heat, entropy change under isothermal magnetization/demagnetization, temperature change under adiabatic magnetization/demagnetization, refrigerant capacity, and magnetic Grüneisen ratio, are calculated and discussed in the Section 3. Section 4 outlines the final remarks.

## 2. Theoretical Model and Its Solution

The present section contains the description of V12 molecular magnet and the model that we use to characterize its low-temperature magnetic properties. The formalism used to describe the thermodynamic properties and the quantities relevant for the description of MCE are derived and discussed.

### 2.1. Theoretical Low-Temperature Model of V12 and Its General Thermodynamics

**Figure 1:**
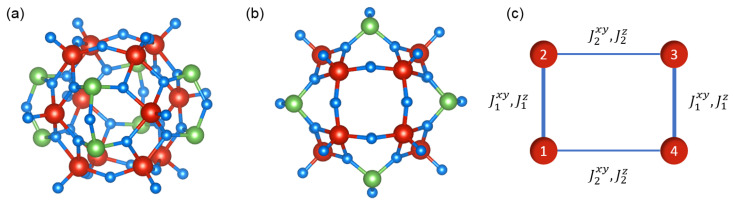
A schematic side view (**a**) and top view (**b**) of the V12 cluster—V atoms marked with red, As atoms with green, O atoms with blue, without the water molecule encapsulated inside for clarity; (**c**) a central tetramer composed of V atoms with spin labels and with the spin-spin exchange couplings marked with lines. The schemes (**a**,**b**) were prepared using VESTA [57] on the basis of crystallographic data for V12 acquired from [58].

The unit cell of V12 compound consists of a [V${}_{12}$As${}_{8}$O${}_{40}$(H${}_{2}$O)]${}^{4-}$ ion which can be considered as isolated, owing to weak interactions with other clusters. The compound that is abbreviated as V12 crystallizes in monoclinic structure (space group P2${}_{1}$/n) with the lattice parameters of a=1215.90 pm, b=2122.99 pm, and c=1370.63 pm and angle $\beta =111.778^{\circ }$ with Z=2 (all data taken from Ref. [13]). Figure 1 shows the schematic picture of the V12 structure as a side view (Figure 1a) and a top view (Figure 1b). Let us note here that, in both figures, solely the cluster V${}_{12}$As${}_{8}$O${}_{40}$, without the guest water molecule inside, is shown for clarity. The detailed structural discussion can be found in Ref. [13]. In both figure panels, a presence of three vanadium ion tetramers is seen, forming a stacked structure with the largest tetramer inside. The magnetic properties of V12 at low temperatures (below approximately 100 K) can be reproduced well when only four spins of V ions from this inner tetramer are taken into account [13]. Figure 1c shows the view of this particular magnetic subsystem with schematically marked magnetic interactions between the spins.

The following anisotropic Heisenberg model is used in order to describe the properties of a single tetramer:

(1) $$\begin{array}{cc} H & =-J_{1}^{xy}\left(S_{1}^{x}S_{2}^{x}+S_{1}^{y}S_{2}^{y}+S_{3}^{x}S_{4}^{x}+S_{3}^{y}S_{4}^{y}\right)-J_{1}^{z}\left(S_{1}^{z}S_{2}^{z}+S_{3}^{z}S_{4}^{z}\right)\\ \; & -J_{2}^{xy}\left(S_{1}^{x}S_{4}^{x}+S_{1}^{y}S_{4}^{y}+S_{2}^{x}S_{3}^{x}+S_{2}^{y}S_{3}^{y}\right)-J_{2}^{z}\left(S_{1}^{z}S_{4}^{z}+S_{2}^{z}S_{3}^{z}\right)-g\mu_{B}B\left(S_{1}^{z}+S_{2}^{z}+S_{3}^{z}+S_{4}^{z}\right). \end{array}$$

In the above Hamiltonian, g=1.97 is the gyromagnetic factor for V ions (after Refs. [13,59]), $\mu_{B}$ is the Bohr magneton, and *B* stands for the external magnetic field along *z* direction in spin space. The quantum operators $S_{i}^{\alpha }$ denote α=x,y,z components of spin S=1/2 localized at the site labelled with i=1,2,3,4. The exchange integrals with the values of $J_{1}^{xy}/k_{B}=-$18.6 K, $J_{1}^{z}/k_{B}=-$19.0 K, $J_{2}^{xy}/k_{B}=-$15.6 K, and $J_{2}^{z}/k_{B}=-$16.0 K are accepted. They are taken from Refs. [13,22], where the inelastic neutron scattering experiment was performed at the temperature of 1.9 K, leading to the precise determination of the energy levels of the magnetic Hamiltonian for the cryogenic range of temperatures, including the interaction anisotropy-induced splittings. The dependence of the exchange coupling integrals on the interatomic distance should not be important for the discussed range of temperatures. All of the couplings are antiferromagnetic in character, with slightly unequal values and also weak spin-space anisotropy of XXZ model type. In the rectangular tetramer geometry, such couplings do not lead to magnetic frustration. Let us comment here that some theoretical models use the simplified Hamiltonian with fully isotropic and identical couplings [23] based on the fitting of temperature dependence of the magnetic susceptibility (the method is less sensitive to the anisotropies of exchange couplings than inelastic neutron scattering).

The full thermodynamic description of the system of interest is constructed on the basis of canonical ensemble (actually using its variant called magnetic field ensemble [60], as the external magnetic field *B* is directly incorporated into the Hamiltonian H). Within this approach, the density operator describing the thermal quantum state is given by

(2) $$\rho =\frac{1}{Z}e^{-H/\left(k_{B}T\right)},$$

where $k_{B}$ is the Boltzmann constant. The statistical sum Z is expressed by:

(3) $$Z=\mathrm{Tr}\;e^{-H/\left(k_{B}T\right)}=\sum_{k=1}^{N}e^{-E_{k}/\left(k_{B}T\right)},$$

with $N=2^{4}$ being a number of states in the Hilbert space of the system of four spins S=1/2 and $E_{k}$ standing for the eigenvalues of the Hamiltonian H.

The Gibbs free energy of a single V12 tetramer is given by

(4) $$G=-k_{B}T\ln Z$$

whereas the expression for free enthalpy yields:

(5) $$H=\mathrm{Tr}\;\left(\rho H\right).$$

Let us comment here that the external field is included in the Hamiltonian, so that the average value of the quantum observable H is free enthalpy, not internal energy of the system in question.

From the point of view of MCE properties, the vital thermodynamic quantity is the magnetic entropy, which can be defined as

(6) $$S=-{\left(\frac{\partial G}{\partial T}\right)}_{B}$$

and conveniently calculated from:

(7) $$S=\frac{H-G}{T}.$$

Another quantity of interest is the magnetic specific heat

(8) $$c_{B}=T{\left(\frac{\partial S}{\partial T}\right)}_{B},$$

which can be calculated from the following convenient formula:

(9) $$c_{B}=\frac{\mathrm{Tr}\;\left(\rho H^{2}\right)-{\left[\mathrm{Tr}\;\left(\rho H\right)\right]}^{2}}{k_{B}T^{2}}.$$

Here, let us emphasize that we focus our interest on the thermodynamic properties of V12, especially in the low-temperature range (including the cryogenic range, where we expect the emergence of the most useful phenomena). Consequently, we construct the description of the thermodynamics including only the purely magnetic contribution. In general, the entropy of the system (and also the specific heat) contains the contribution coming from the purely magnetic subsystem of interacting, localized magnetic moments (originating here from the localized *d*-type electronic states of V atoms), from the lattice vibrations and from the charge carriers.

In the range of low temperatures, especially the cryogenic ones, the leading contribution to the total entropy (and to the specific heat) is expected to come entirely from the magnetic degrees of freedom. This is due to the fact that the energy scales that are involved in magnetic exchange for the cluster molecular magnets are usually low compared to the characteristic energies of lattice vibrations. It can be detected in the experimental data for specific heat, such as those reported for V15 [61] or, for other example compounds, in Refs. [62,63]. In addition, the entropy component that originates from the lattice vibrations is, to a large extent, independent on the applied magnetic field. This feature enables the usual treatment of experimental data on low-temperature specific heat for molecular magnets, when the Einstein model-based expression [64] is fitted to the difference of zero-field and high-field data (assumed to be the lattice contribution), as, for example, in Ref. [61].

In the particular case of V12, we should mention that the compound is composed of weakly interacting clusters. The cluster vibrational energies have been calculated in Ref. [14] by the DFT method and for V12 polyoxovanadates of V${}_{8}^{\mathrm{IV}}$V${}_{4}^{V}$ type the lowest energies are over 13 meV (which corresponds to the thermal energy $k_{B}T$ at over 150 K), so the lattice vibrations should not make a significant contribution in the cryogenic temperature temperature range. As a consequence, it appears justified to disregard the lattice subsystem for the purpose of characterization of the low-temperature magnetocaloric properties and focus the attention solely on the spin system (see also the discussion in the Section 4).

Regarding the charge carriers contribution to the system entropy, we note that, according to Ref. [14], V12 shows a significant energy gap that exceeds 1 eV (as shown by the experiment and supported by DFT calculations). Therefore, the contribution of the thermally excited charge carriers to the total entropy should not be significant at low temperatures, which justifies neglecting this component of the total entropy and specific heat of the system.

The typical DFT approach to molecular magnets consists in predicting the exchange integral values in order to provide parameters for localized spin model Hamiltonian (of anisotropic Heisenberg type). In this context, a work concerning V15 polyoxovanadate cluster with magnetic ions arranged in triangle and hexagons can be mentioned [65,66,67]. The predicted values allow for the further analysis of the purely magnetic properties, basing on the Heisenberg Hamiltonian. However, the complete description of the system thermodynamics at wide range of temperatures, including high ones, would call for extensive computational modelling of the properties, including the energy scales of lattice vibrations as well as the charge carrier excitations. In the present work, we focus our attention on the low-temperature range, so that such an ambitious goal is not addressed.

Finally, let us mention the literature example in which a purely magnetic model has been successfully used to reproduce the experimental results concerning the single-molecule MCE in Gd-based cluster [68].

In view of the above discussion, we proceed with characterizing the low-temperature magnetocaloric properties of V12 by using a model that only involves the magnetic degrees of freedom.

### 2.2. Thermodynamic Quantities of Interest from the Magnetocaloric Point of View

On the basis of the above-mentioned formalism, the thermodynamic quantities of interest from the magnetocaloric point of view can be calculated and discussed [69].

In order to quantify the magnetocaloric properties of the system, the most crucial quantity is the isothermal entropy change $\Delta S_{T}$, i.e., the change in system entropy when the external magnetic field is varied between some initial value B>0 and the final value of B=0. Thus, it is defined by:

(10) $$\Delta S_{T}=S\left(T,B=0\right)-S\left(T,B\right).$$

Within the accepted convention, the positive value, $\Delta S_{T}>0$, corresponds to the direct MCE (entropy decreases after applying the field B>0), whereas $\Delta S_{T}<0$ describes the inverse MCE (when entropy is increased after applying the field B>0). The isothermal entropy change can be directly related to the specific heat, namely:

(11) $$\Delta S_{T}=\int_{0}^{T}\frac{c_{B}\left(T^{\prime },B=0\right)}{T^{\prime }}\;dT^{\prime }-\int_{0}^{T}\frac{c_{B}\left(T^{\prime },B\right)}{T^{\prime }}\;dT^{\prime },$$

where such relation can be used to derive $\Delta S_{T}$ from the experimental data.

A quantity describing the performance of a particular magnetocaloric material in a process that occurs between the constant temperatures $T_{1}$ and $T_{2}>T_{1}$ can be the refrigerant capacity defined as [30,70,71]:

(12) $$\Delta Q_{RC}=\int_{T_{1}}^{T_{2}}\Delta S_{T}\;dT.$$

This formula yields the amount of heat transferred between the reservoirs in a single refrigeration cycle, for arbitrary selection of the temperatures $T_{1}$ and $T_{2}$.

It is rather common to consider the behaviour of isothermal entropy change in the vicinity of a local extremum, like one that is related to the phase transition or, in our case, to the presence of quantum level crossing. The refrigerant capacity $\Delta Q_{RC}^{*}$ can be defined in such a case by integrating the isothermal entropy change over a range of temperatures covering the full width at half maximum of the peak of $\Delta S_{T}$. Namely, the limiting temperatures are selected according to the condition $\Delta S_{T}\left(T_{1}\right)=\Delta S_{T}\left(T_{2}\right)=\frac{1}{2}\Delta S_{T}\left(T_{max}\right)$, where, at $T_{max}$, the isothermal entropy change takes the local extremum. This parameter provides a single value that is commonly used to characterize the performance of the material and compare it to other materials, but it should be stressed that the range of temperatures varies if the magnitude of magnetic field variation is changed.

All of the calculations based on the entropy change in isothermal process can be expected to be valid for general low-temperature range, since the potential lattice contribution to the entropy should be only temperature-dependent and only weakly magnetic field-dependent. Therefore, the model neglecting the lattice or charge carrier degrees of freedom should be fully valid for the calculation of the isothermal entropy change and related quantities.

Under adiabatic conditions, the response of the magnetic material to the magnetic field variation is the change in its temperature. This manifestation of MCE can be quantified by the adiabatic temperature change, $\Delta T_{S}$, also being a fundamental magnetocaloric quantity of interest and determined from the equation:

(13) $$S\left(T,B_{i}\right)=S\left(T-\Delta T_{S},B_{f}\right),$$

when the initial temperature is equal to *T* and the field is varied between $B_{i}$ and $B_{f}$ value. Let us note that from a practical point of view, to achieve cooling effect, the positive values of $\Delta T_{S}$ should be obtained for a given selection of the initial and final values of the magnetic field. Therefore, for direct MCE, a natural selection is $B_{i}=B$ and $B_{f}=0$, whereas for inverse MCE $B_{i}=0$ and $B_{f}=B$ lead to the temperature decrease.

Once more, it has to be emphasized here that the total entropy of the magnetic system consists not only of the purely magnetic contribution, but also includes components that originate from the lattice vibrations or from the charge carriers. These mentioned non-magnetic contributions are usually dependent mainly on the temperature and their isothermal variation with the magnetic field is typically weak (unless, for example, structural phase transitions are involved). Consequently, in the case of isothermal entropy change, the purely magnetic component strongly dominates over the remaining components. The situation is quite different for the adiabatic temperature change, when both temperature and magnetic field are changed in the adiabatic process. For this process, the usage of only magnetic entropy gives an estimate of the real temperature change, but in general it tends to be overestimated (as all the entropy components can be expected to increase with the increasing temperature). However, in view of the discussion in the previous subsection, the other entropy components in V12 (lattice and charge carrier-related) should not contribute significantly in the range of cryogenic temperatures. Therefore, the model used should be reliable for the calculation of the adiabatic temperature change for the range of cryogenic temperatures.

The isothermal entropy change or adiabatic temperature change are the quantities that are appropriate for finite field change, giving a global description of the magnetocaloric response of the system. To supplement this picture, a local quantity can be used, called a magnetic Grüneisen ratio. It can be defined as:

(14) $$\Gamma_{B}=-\frac{1}{c_{B}}{\left(\frac{\partial S}{\partial B}\right)}_{T}$$

and it quantifies the local response of the magnetic entropy to the differential change of the field. On the other hand, while using the equilibrium thermodynamic identities, it can be alternatively expressed as:

(15) $$\Gamma_{B}=\frac{1}{T}{\left(\frac{\partial T}{\partial B}\right)}_{S},$$

which form describes the local temperature response to magnetic field change under adiabatic conditions.

The magnetic Grüneisen parameter, being a local quantity, can be related to the isothermal entropy change by using the following formula:

(16) $$\Delta S_{T}=\int_{0}^{B}c_{B}\left(T,B^{\prime }\right)\Gamma_{B}\left(T,B^{\prime }\right)\;dB^{\prime },$$

while the adiabatic temperature change can be calculated from the relation:

(17) $$\Delta T_{S}=\int_{0}^{B}T\;\Gamma_{B}\left(T,B^{\prime }\right)\;dB^{\prime }.$$

The presented formulas allow the extensive calculation of the crucial thermodynamic quantities of interest for the characterization of MCE within the field ensemble formalism. The calculation is numerically exact when the Hamiltonian given by Equation (1) is diagonalized with the numerically exact method. All of our further calculations of the thermodynamic quantities presented in the next section were performed in this manner, by using Wolfram Mathematica software [72].

## 3. Numerical Results and Discussion

The current section contains a presentation of the numerical calculations focused at characterization of the thermodynamics of V12 from the magnetocaloric point of view. The extensive quantities (entropy, specific heat, refrigerant capacity, and isothermal entropy change) are expressed per mole of V12, not per a single cluster, for convenience.

**Figure 2:**
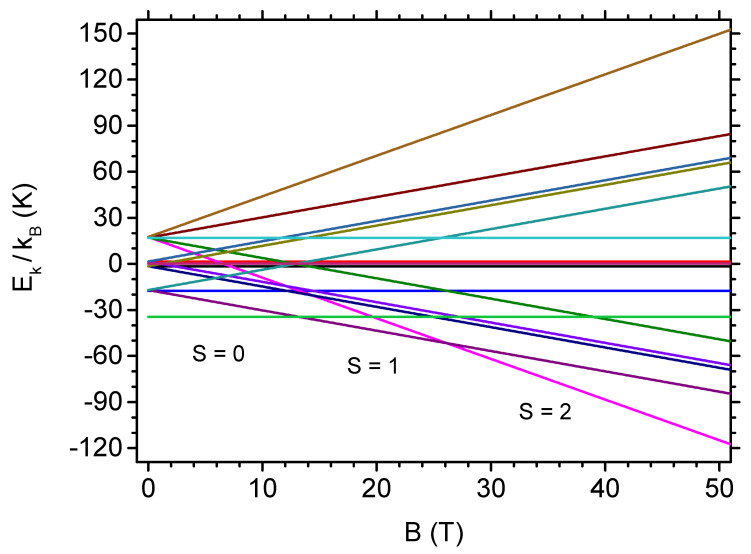
The dependence of the normalized eigenenergies of the tetramer Hamiltonian on the external magnetic field. The values of ground-state spin, S=0,1,2 are marked below the ground state.

Let us commence the analysis from the presentation of the energy spectrum of the tetramer Hamiltonian given by Equation (1) and its dependence on the external magnetic field. Figure 2 shows such dependence for all 16 eigenenergies (the eigenenergies $E_{k}$ are normalized by $k_{B}$ to express them in temperature units for convenience). It is visible that, in the absence of the external magnetic field, the unique ground state of the tetramer has spin S=0. The state with S=0 remains the ground state up to the critical field $B_{c,1}=$ 13.3 T, at which quantum level crossing occurs and, for $B>B_{c,1}$, the ground state has S=1. The next quantum level crossing takes place at $B_{c,2}=$ 26.1 T, where the system achieves magnetic saturation, i.e., the spin takes the maximum value of S=2. The energies of all three mentioned states and values of the critical fields at which quantum level crossings occur are discussed in details for our anisotropic tetramer in the Appendix A. It should be mentioned that the presence of accidental two-fold ground state degeneracies at $B_{c1,}$ and at $B_{c,2}$ would give rise to residual entropy exactly at that values of the external magnetic field and would strongly manifest itself in further discussion of the magnetocaloric properties of V12.

Here, it can be noted that the exact form of the energy spectrum of V12 molecular magnet at B=0 has attracted considerable attention and it has been accessed by using an inelastic neutron scattering experimental approach to explain its detailed structure [13].

**Figure 3:**
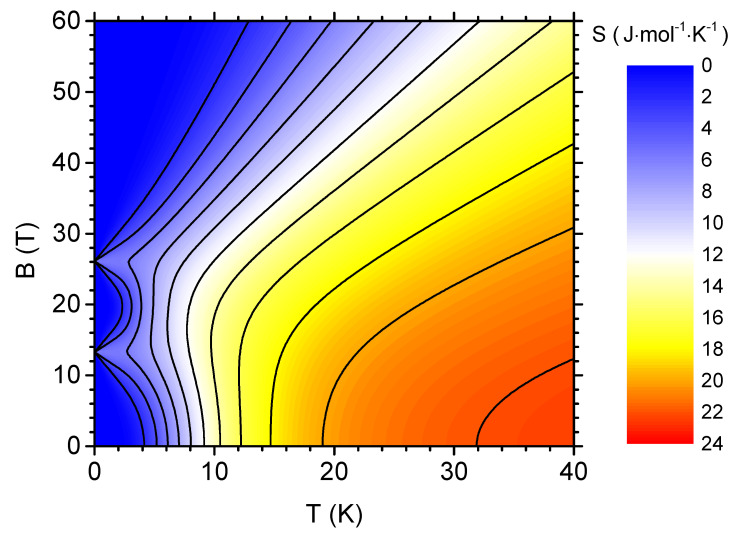
The density plot with contours showing the magnetic entropy per mole as a function of the temperature and the external magnetic field.

The fundamental thermodynamic quantity of interest from the magnetocaloric point of view is the magnetic entropy. Figure 3 presents the magnetic entropy of V12 (expressed per mole) as a density plot with contour lines (isentropes) in temperature-magnetic field plane. The density plot shows the traces of the quantum level crossings at $B_{c,1}$ and $B_{c,2}$: exactly at the critical magnetic fields the residual entropy equals to S=Rln2≃0.693R≃5.76 J·mol${}^{-1}\cdot$K${}^{-1}$, whereas it is equal to 0 otherwise. The shape of the isentropes indicates that, at finite temperatures, the presence of quantum level crossings gives rise to two distinct entropy peaks when the magnetic field is changed under isothermal conditions. When the temperature increases, both maxima tend to merge what takes place finally below 10 K. Above the temperature of approximately 12 K, the entropy tends to decrease monotonically as a function of the magnetic field for constant temperature. It can be concluded that the form of isentropes in the vicinity of both critical fields predicts the inverse MCE, possible at low temperatures and fields that are close to the critical values. In the remaining area of the diagram, the direct MCE would be expected, as the entropy decreases when the field is applied.

**Figure 4:**
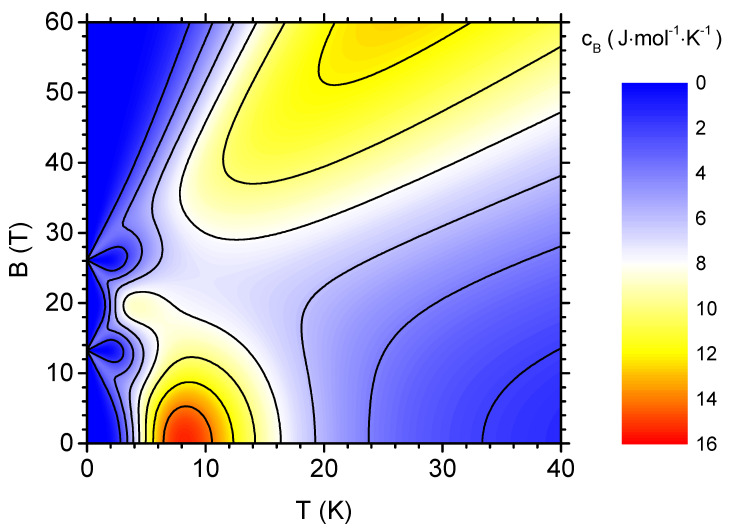
The density plot with contours showing the magnetic specific heat per mole as a function of the temperature and the external magnetic field.

The quantity closely connected with the magnetic entropy and important for the magnetocaloric response is the magnetic specific heat (for constant external magnetic field). Figure 4 shows the specific heat of V12 per mole as a density plot with contour lines, in the temperature-magnetic field plane. When the behaviour of $c_{B}$ in the absence of the external magnetic field is analysed, a pronounced peak located around 9 K attracts the attention. This maximum is reduced if the magnetic field is applied, but its position remains unshifted. In the vicinity of the critical fields $B_{c,1}$ and $B_{c,2}$, distinct features are observable in the specific heat, reflecting the behaviour of the magnetic entropy in this range of parameters. Namely, when the magnetic field is increased at low temperature, a double maximum (slightly below and slightly above the critical field) is passed. Exactly at the critical field, a robust minimum is reached. In particular, close to the second critical field, the specific heat remains low in the whole depicted range of temperatures. In the range of high magnetic fields, above the second critical field, a maximum at high temperatures appears and its position is shifted to higher temperatures when the field increases.

**Figure 5:**
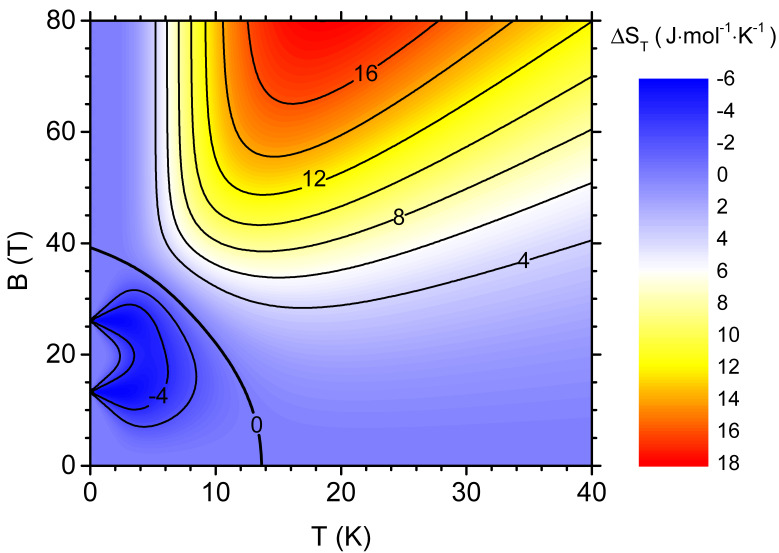
The density plot with contours showing the isothermal entropy change per mole as a function of the temperature and the initial value of the external magnetic field in the isothermal process (the final value of the magnetic field is zero).

The magnetocaloric response of the system due to field variation between the initial non-zero value *B* and final zero value can be globally quantified with isothermal entropy change $\Delta S_{T}$, defined in Equation (10). Figure 5 shows the value of the isothermal entropy change per mole as a density plot with additional contour lines in the plane temperature-initial magnetic field *B*. In the convention used, positive values correspond to direct MCE, whereas negative ones indicate the inverse MCE. In the plot Figure 5 both ranges can be identified, separated by parabolic-like boundary of $\Delta S_{T}=0$. The inverse MCE is predicted for temperatures lower than approximately 14 K and this range of temperatures is reduced by the increasing magnetic field amplitude. For the field amplitudes above 40 T, direct MCE is predicted for the whole range of temperatures. The most pronounced inverse MCE (with two separate minima) is predicted when the initial magnetic field is close to one of the two critical fields. If the temperature is slightly increased, both minima merge into single wider extremum. It should be noticed that the most pronounced inverse MCE can occur approximately at liquid helium temperatures, close to 4 K, for the initial fields between 10 and 30 T. Direct MCE is predicted for either higher temperatures or higher magnetic fields, with a maximum at approximately 20 K. The knowledge of the density plot of $\Delta S_{T}$ permits the identification of the most interesting ranges of temperature and magnetic field for desired magnetocaloric behaviour.

**Figure 6:**
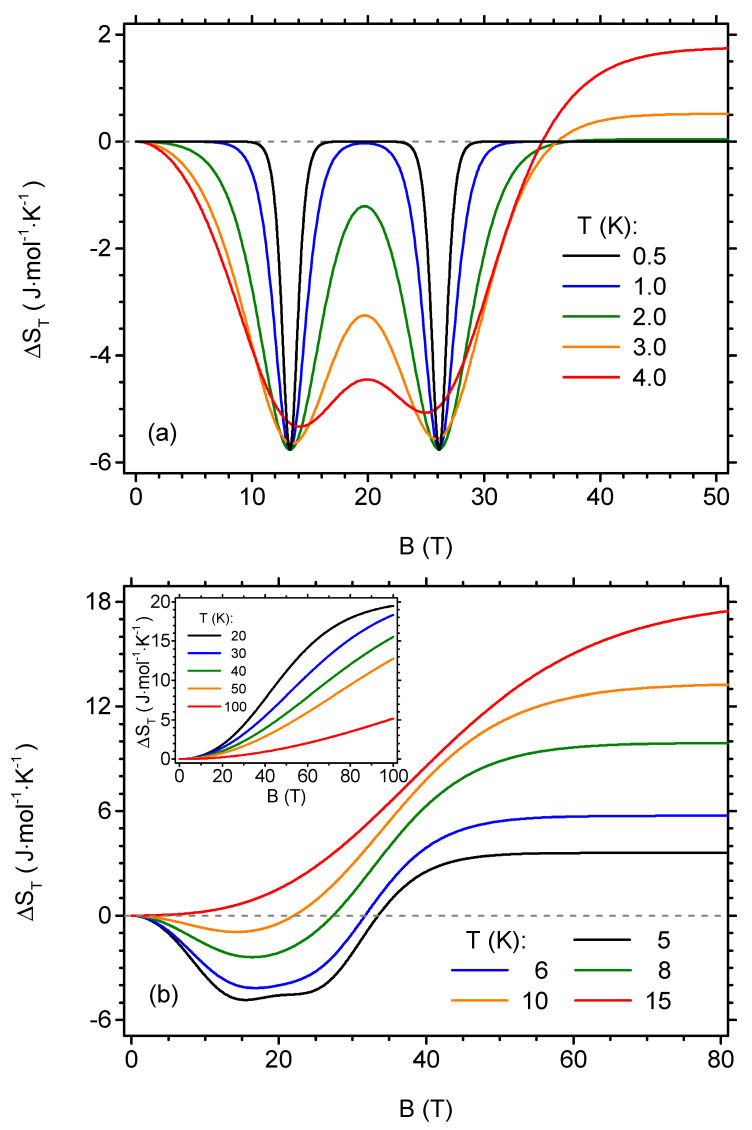
The dependence of the isothermal entropy change per mole on the initial value of the external magnetic field in the isothermal process (the final value of the magnetic field is zero), for selected temperatures. (**a**) low-temperature range; (**b**) higher temperature range.

The cross-sections of the plot shown in Figure 5 can be studied to gain more insight into the isothermal entropy change behaviour. First, the dependence of $\Delta S_{T}$ on the initial value of the magnetic field can be investigated, for several representative temperatures. Such analysis is possible on the basis of Figure 6. The plot Figure 6a presents the data for the lowest temperatures, T≤ 4 K. At very low temperature, like 0.5 K, two narrow minima of $\Delta S_{T}$ corresponding to the critical field values are visible when the initial field is changed. The depth of each minimum (i.e., the amplitude of entropy change) is $|\Delta S_{T}|=R\ln2≃0.693R≃$ 5.76 J·mol${}^{-1}\cdot$K${}^{-1}$. This is due to the already discussed fact that, exactly at critical field, the quantum level crossing occurs and the ground state is two-fold degenerate. When the temperature increases, the minima get wider, but their depth is essentially unchanged below 3 K. For 4 K, the clear tendency for merging of both minima becomes evident and also a slight asymmetry arises, as the minimum for lower critical field becomes deeper than the one for higher critical field. Below 2 K, the direct MCE at higher magnetic fields is very much reduced in magnitude, but it gets more significant when *T* > 2 K. Figure 6b depicts further behaviour of isothermal entropy change for *T* > 4 K. In its main panel, it is visible how both minima merge into a single wide minimum which becomes increasingly shallow when the temperature is increased. Finally, for *T* > 15 K, only the direct MCE occurs in the whole range of fields. It is also visible that in the range of direct (positive) MCE, the magnitude of $\Delta S_{T}$ becomes saturated as a function of the field. Figure 6b shows the influence of the highest studied temperatures on the $\Delta S_{T}$ in the inset, where the monotonical dependence of isothermal entropy change on the initial magnetic field is visible and the mentioned tendency to saturation is no longer noticeable for the investigated range of magnetic fields.

**Figure 7:**
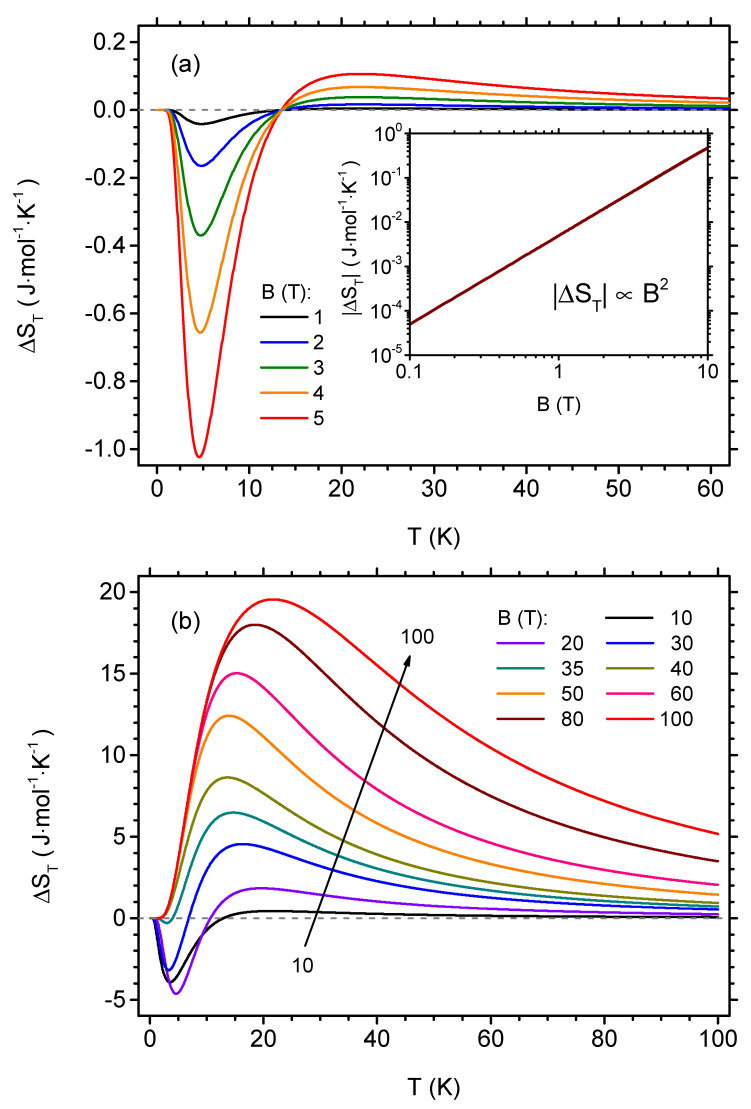
The dependence of the isothermal entropy change per mole on the temperature, for selected initial values of the external magnetic field in the isothermal process (the final value of the magnetic field is zero). (**a**) low-field range; (**b**) higher field range. The inset in (**a**) shows the absolute value of isothermal entropy change as a function of the initial value of the external magnetic field for the local minimum at approximately 5 K in double logarithmic scale.

Another interesting cross-section of Figure 5 is the dependence of the isothermal entropy change on the temperature for some fixed initial magnetic fields, as shown in Figure 7. The plot in Figure 7a presents the results obtained for the initial fields B≤ 5 T, for which the inverse MCE is most pronounced at low temperatures. A single minimum around 4.6...4.8 K is evident; moreover, it can be noticed that the dependence of $|\Delta S_{T}|$ on *B* in this minimum is non-linear. The plot in double logarithmic scale shown in the inset in Figure 7a demonstrates that for this minimum the quadratic dependence of $|\Delta S_{T}|$ on the initial field value holds. This behaviour contrasts with the usually noticed linear dependence of isothermal entropy change on the field. For the low field range, the temperature at which the minimum occurs is only very weakly dependent on the field. When the field is increased, a maximum of direct MCE builds up for higher temperatures (and its position is moving when the field is changed—first, it shifts towards higher temperatures and then it moves back). The behaviour of $\Delta S_{T}$ for higher initial field can be followed in Figure 7b. When *B* > 20 T, the low-temperature minimum of inverse MCE tends to get more shallow and eventually vanishes for *B* > 35 T. Subsequently, only a single, pronounced maximum is present, around 20 K. For this maximum in the high-field range, the dependence of $\Delta S_{T}$ on the initial field becomes finally sublinear.

**Figure 8:**
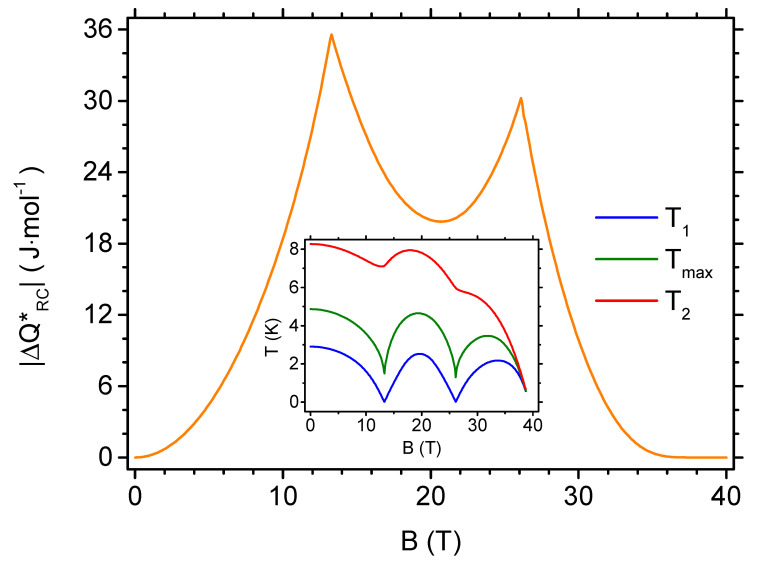
The dependence of the absolute value of refrigerant capacity per mole on the initial value of the external magnetic field in the isothermal process (the final value of the magnetic field is zero), for temperatures between $T_{1}$ and $T_{2}$, covering the full width at half maximum of the entropy change peak. The inset shows the dependence of the limiting temperatures $T_{1}$ and $T_{2}$ as well as the temperature $T_{max}$ at which entropy change reaches the extremum on the initial value of the external magnetic field.

Let us now follow the dependence of the refrigerant capacity $\Delta Q_{RC}^{*}$ on the magnitude of the magnetic field variation in order to analyse the magnetocaloric performance in processes with temperature variation. Let us remind that this quantity results from the integration of the isothermal entropy change over the range of temperatures corresponding to full width at half maximum of the entropy change peak according to Equation (12). In our study, we focus the attention on the range of inverse MCE with well pronounced local extremum of $\Delta S_{T}$ in the cryogenic range. For convenience, we plot the absolute value of $\Delta Q_{RC}^{*}$, because $\Delta S_{T}$ is negative for inverse MCE according to the definition given by Equation (10). Figure 8 shows the relevant results. A structure with two maxima is visible (corresponding to the critical fields for quantum level crossings). Below the first critical field, a quadratic dependence of $Q_{RC}^{*}$ on the magnetic field is present. The range of temperatures covering the full width at half maximum of the peak varies in general with the magnetic field variation magnitude, as has been mentioned in Section 2. To visualise this sort of behaviour, we added an inset to Figure 8, showing the limiting temperatures of the integration range, $T_{1}$ and $T_{2}$, supplemented with the temperature $T_{max}$ at which the entropy change achieves an extremum. The characteristic temperature $T_{max}$ is shifted towards $T_{1}$ due to asymmetry of the peak of the isothermal entropy change. For all of the considered magnetic fields, the range of integration remains in the cryogenic regime.

**Figure 9:**
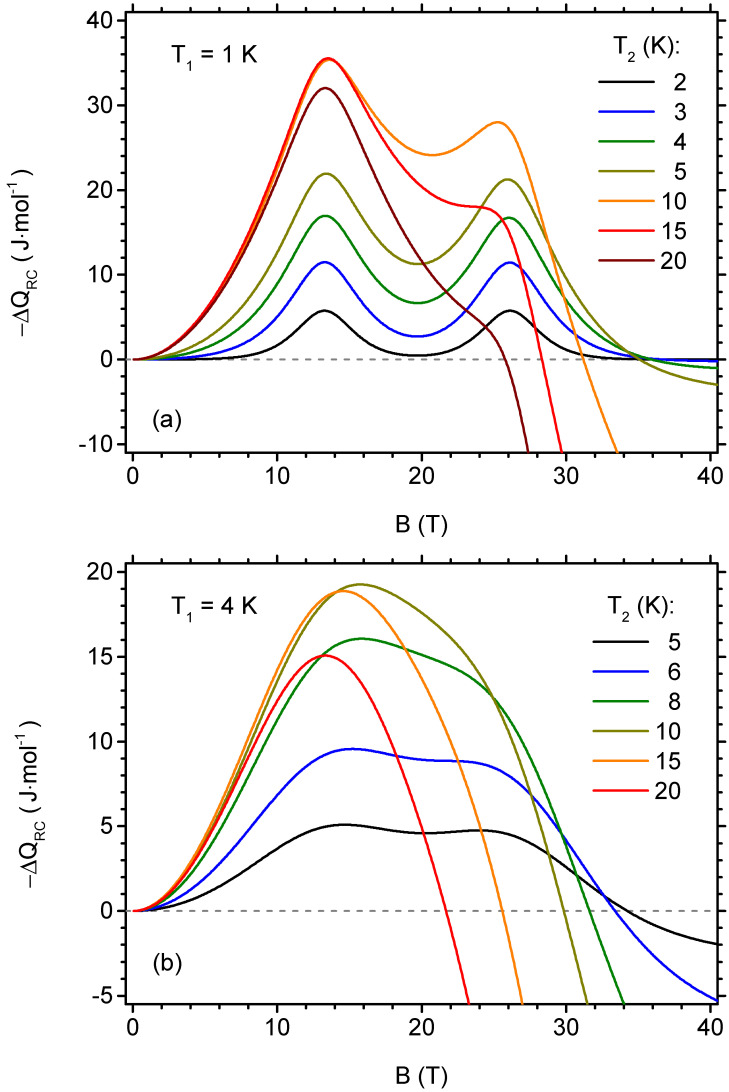
The dependence of the refrigerant capacity per mole on the initial value of the external magnetic field in the isothermal process (the final value of the magnetic field is zero), for selected fixed temperature ranges, with initial temperature $T_{1}$ of 1 K (**a**) and 4 K (**b**), for selected final temperatures $T_{2}$.

We additionally analyse the behaviour of the refrigeration capacity $\Delta Q_{RC}$ defined between fixed temperatures $T_{1}$ and $T_{2}$ in order to sketch the magnetocaloric performance for the fixed ranges of working temperatures (given by Equation (12)). Again, we focus our interest on the range of low temperatures with inverse MCE, for which we present the results in Figure 9, selecting the lower limiting temperature as 1 K in Figure 9a and 4 K in Figure 9b and choosing the higher limiting temperatures up to 20 K. Again, the $-\Delta Q_{RC}$ is plotted in order to obtain positive values for inverse MCE for convenience. For the first case, the pronounced behaviour with two extrema is seen for the higher temperature $T_{2}<$15 K, while for wider range of temperatures only the low-field maximum tends to survive. Again, for the fields below the first critical field, the refrigeration capacity increases quadratically with the applied field. It is seen that $-\Delta Q_{RC}$ becomes negative for *B* strong enough, which is a trace of switching between inverse and direct MCE. For the lower limiting temperature of 4 K, a robust single maximum is seen and the change to direct MCE takes place at lower magnetic fields.

**Figure 10:**
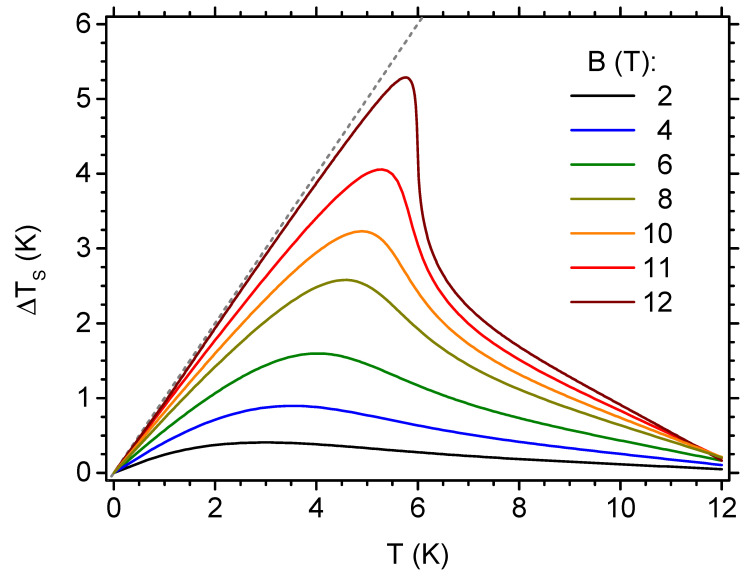
The dependence of the adiabatic temperature change on the initial temperature, for selected final values of the external magnetic field in the isothermal process (the initial value of the magnetic field is zero). The dashed line shows the limiting value of the adiabatic temperature change, with a final temperature equal to 0.

Another quantity characterizing the MCE is adiabatic temperature change, as defined by Equation (13). We present the predicted behaviour of this quantity in Figure 10, again solely focusing the attention on the range of inverse MCE. In the plot, the adiabatic temperature change is shown as a function of the initial temperature for selected values of the final magnetic field (whereas the initial field is set to 0, in order to achieve temperature decrease for inverse MCE). The dashed line shows the theoretical absolute limit of temperature drop, which is equal to $\Delta T_{S}=T$. It can be observed that the adiabatic temperature change develops a single asymmetric peak. Particularly high values of temperature change can be predicted when using the fields close to the lower critical field $B_{c,1}$, for the initial temperature around 6 K. It should be stressed here that our theoretical model only involves the purely magnetic degrees of freedom, not taking into account the other components of the total entropy of the system (e.g., the contribution of the lattice/cluster vibrations—see the remark in Section 2 on the vibrational energies calculated in Ref. [14]). Therefore, the values of adiabatic temperature change that are given in Figure 10 should be treated as upper limits. However, for the range of the lowest temperatures, the magnetic entropy can constitute a leading contribution to the total entropy of the system (see, for example, the experimental data for V15 polyoxovanadate in Ref. [61], where the magnetic contribution to the specific heat is dominant below 4 K).

A local quantity can be investigated in order to supplement the global picture of MCE response, provided by the analysis of the isothermal entropy change and adiabatic temperature change for finite field intervals.

**Figure 11:**
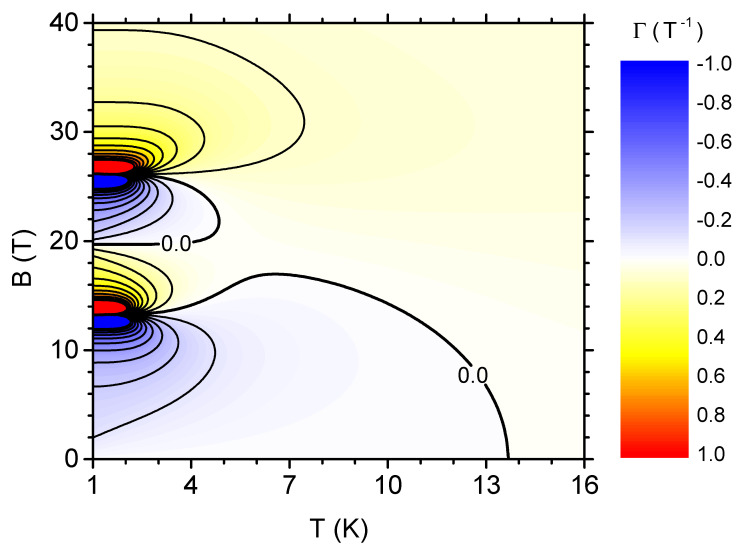
The density plot with contours showing the magnetic Grüneisen ratio as a function of the temperature and the external magnetic field.

The local response of the magnetic entropy of the system to the change of magnetic field can be captured with magnetic Grüneisen ratio, as defined by Equation (14). Figure 11 presents a density plot with contours for this quantity as a function of the temperature and external magnetic field. The ranges of both positive and negative $\Gamma_{B}$ are visible, corresponding to locally direct or inverse MCE. In particular, for fields that were approximately lower than the first critical field $B_{c,1}$ for temperatures lower than 13 K, we have negative values of the magnetic Grüneisen ratio, corresponding to locally inverse MCE. Another such range is for temperatures below 4 K, for the fields belonging to the upper half of the segment between $B_{c,1}$ and $B_{c,2}$. For the remaining area of the plot (especially for strong fields and/or high temperatures), the magnetic Grüneisen ratio is positive, indicating locally direct MCE. It should be noticed that the ranges do not fully overlap with the ranges that are shown in Figure 5, showing the isothermal entropy change, being a global quantity and resulting from the integration of Grüneisen ratio according to Equation (16).

**Figure 12:**
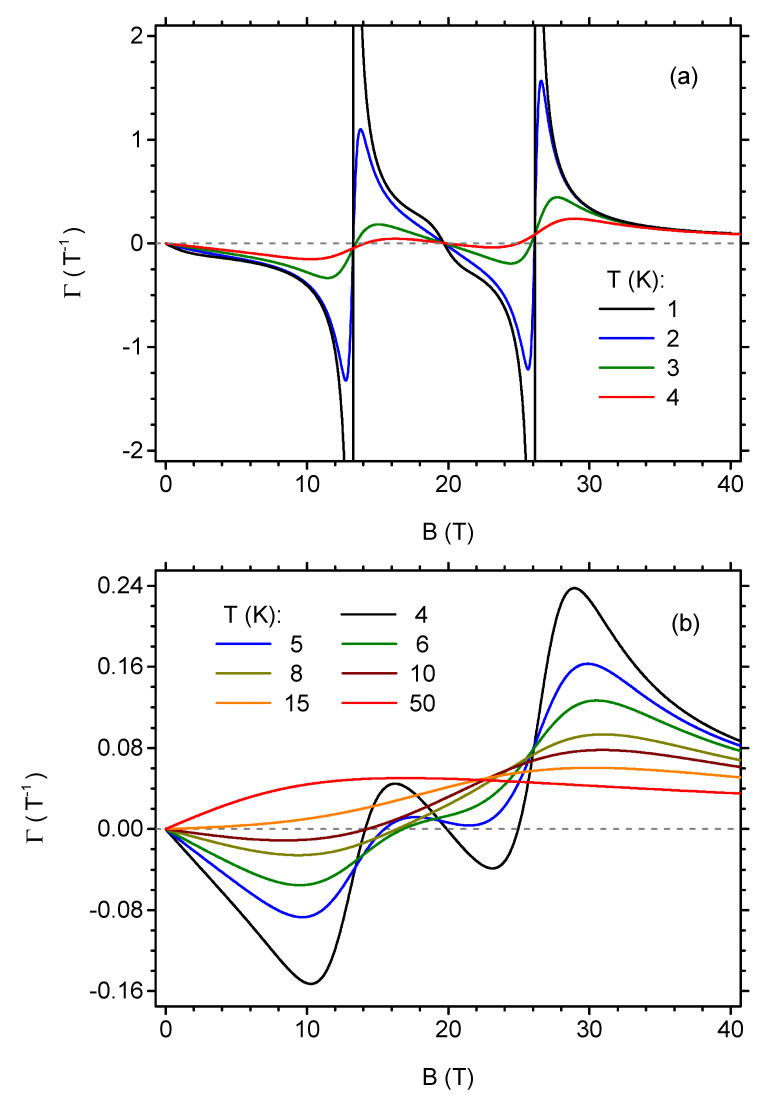
The dependence of the magnetic Grüneisen ratio on the initial value of the external magnetic field in the isothermal process (the final value of the magnetic field is zero), for selected temperatures. (**a**) low-temperature range; (**b**) higher temperature range.

It is remarkable that $\Gamma_{B}$ takes the largest absolute values in the close vicinity of both critical fields (negative values slightly below and positive values slightly above the given critical field). It can be particularly instructive to study the cross-sections of the plot shown in Figure 11 for constant temperatures. Such data are presented in Figure 12, where $\Gamma_{B}$ is plotted as a function of the magnetic field for constant temperatures. Panel Figure 12a is focused on the range of lowest temperatures. Because the magnetic Grüneisen ratio is divergent at each critical magnetic field when T→0, for the lowest but finite temperatures, the dependence shows pronounced minima (maxima) for magnetic fields that are slightly lower (higher) than the critical fields $B_{c,1}$ and $B_{c,2}$. Increasing temperature causes the dependence to flatten; therefore, the range of higher temperatures is separately plotted in Figure 12b. The increasing temperature forces the separate extrema related to each critical magnetic field to diminish; the magnetic Grüneisen ratio takes negative values for lower magnetic fields and positive values for higher fields and just two extrema are seen. Eventually, for T> 14 K, only positive values of $\Gamma_{B}$ are observable in the full range of fields, with a single wide maximum, in consistence with Figure 11.

## 4. Final Remarks

In the paper, we have presented a theoretical prediction of the magnetocaloric properties of V12 polyoxovanadate molecular magnet in the range of temperatures that are below approximately 100 K. In such range, the model that is based on a tetramer composed of spins S=1/2 with weakly unequal and anisotropic interactions described with Heisenberg Hamiltonian captures the thermodynamic properties of the system. The validity of the model (neglecting the lattice and the charge carrier contribution to the entropy) has been extensively justified. The description of the thermodynamics was based on exact analytic and numerical diagonalization of the Hamiltonian and on a variant of canonical ensemble. The fundamental quantities of interest were magnetic entropy, magnetic specific heat, isothermal entropy change, adiabatic temperature change, refrigerant capacity, and magnetic Grüneisen ratio. All of these properties were extensively discussed as a function of the temperature and external magnetic field.

First of all, the crucial influence of the presence of two quantum level crossings on the low-temperature thermodynamics of V12 was predicted. The ground state of V12 in the absence of the field has S=0 (and it is non-degenerate); applying the external field causes the subsequent transitions to states with S=1 and S=2 (magnetic saturation of the tetramer). Let us note that the existence of quantum level crossings has been suggested as a route to the maximization of the magnetocaloric response [41]. Moreover, the quantum level crossings in molecular magnets of non-interacting cluster type have attracted the attention of experimentalists [40,73,74,75]. The significant manifestation of quantum level crossings in the thermodynamic properties can be seen, for example, in the extensive calculations devoted to various model spin trimers [76]. The appropriate values of the critical magnetic fields at which quantum level crossings occur for V12 (approximately 13 T and 26 T) lie within the experimentally accessible range, which could motivate the studies.

Magnetic Grüneisen ratio was one of quantities studied by us, frequently used in the literature to quantify the magnetocaloric local response of various zero-dimensional systems. The magnetic Grüneisen ratio is a quantity of interest not only for characterization of MCE, but also for description of quantum critical points [77,78,79,80]. Let us note that within our model, the system of interest is a finite, zero-dimensional tetramer structure, for which no phase transitions are expected, so that we do not argue presence of quantum critical points but just quantum level crossings at zero temperature. Nevertheless, we predict the divergence of the magnetic Grüneisen ratio at the critical fields for which the quantum level crossings occur. This phenomenon also might serve as a motivation of experimental studies, especially taking into account that some weak intercluster magnetic interactions should exist in V12, which could shape the magnetic properties for the lowest temperatures and hypothetically drive the system towards some sort of quantum criticality.

We find it particularly interesting that the most pronounced inverse magnetocaloric response (the local minimum of isothermal entropy change for finite field variation) occurs in the cryogenic temperature range. This gives the opportunity of cooling by adiabatic application of the magnetic field, rather than by adiabatic demagnetization as in the case of direct MCE in the material. Moreover, the entropy change in the mentioned minimum scales quadratically with the magnetic field variation magnitude. Such a type of variability of entropy change with the magnetic field magnitude has been observed for example in Ref. [81] as an universal behaviour of inverse MCE far from the critical magnetic field; it is also found in Dy compounds [82]. Let us notice that the scaling that was observed by us applies for $B<B_{c,1}$. We can comment here that for direct MCE, the quadratic dependence is found as a high-temperature limiting behaviour [32]. The largest absolute value of the isothermal entropy change in inverse MCE is Rln2≃0.693R≃ 5.76 J·mol${}^{-1}\cdot$K${}^{-1}$ achieved for the temperatures below 3 K for the magnetic field variation magnitude equal to $B_{c,1}$ or $B_{c,2}$, i.e., to approximately 13.3 or 26.1 T. This value of isothermal entropy change results from the presence of quantum level crossings at which the ground state becomes twofold degenerate. As a consequence, we believe that, for V12, the range of inverse MCE is easily accessible experimentally and it might be of importance for cryogenic cooling.

Among the molecular magnetic materials exhibiting the most pronounced MCE in the cryogenic range, various examples can be mentioned [31,34,56]. For instance, Mn-based magnetic sponges show somehow similar values of the isothermal entropy change: it amounts to about 6 to 7 J·mol${}^{-1}\cdot$K${}^{-1}$ for the field of 5 T at temperatures 10 to 25 K, depending on the compound [83]. Additionally, octacyanoniobates containing Ni or Mn exhibit rather similar values of isothermal entropy change below 10 K [84]. Another octacyanoniobate containing Fe exhibits somewhat similar values of the entropy change with a peak slightly above 20 K [85]. In general, various octacyanometallates (both cluster-like and coordination polymers) can be considered to be candidates for cryogenic cooling [32]. Let us note that, in the mentioned molecular magnetic materials, the origin of the isothermal entropy change maximum can be traced back to critical behaviour related to low-temperature magnetic ordering. As a consequence, the linear or sublinear dependence of the isothermal entropy change on the field magnitude is observed, contrary to our predictions of quadratic increase in V12. Moreover, the mentioned compounds show direct MCE. In addition to the above-mentioned compounds, some systems that are based on weakly-interacting clusters can be listed to give a flavour of the experimental results. Among them, Ni-based spin-1 dimer [86] shows the maximum adiabatic temperature change close to 10 K and also a pronounced isothermal entropy change with shifting maximum also in the mentioned range. Maxima of both relevant quantities of interest for MCE can be found approximately at 2 K for Ga-based dimeric system [87]. Layered Ga hydroxides [88], GaF${}_{3}$ [89], or Fe${}_{17}$ [90] cluster also exhibit good cooling properties at few K. For the temperatures below 1 K, Ga-containing polyoxometalates can be mentioned [91]. Last, but not least, experimental studies of V15 polyoxovanadate revealed a maximum isothermal entropy change of about 12.5 J·mol${}^{-1}\cdot$K${}^{-1}$ approximately at 1.5 K for the field variation of 8 T [61].

It can be highlighted that polyoxovanadate structures offer various cluster geometries as a platform for spin-1/2 magnetic systems, which leads to a variety of magnetocaloric properties. In this context, the triangle-based structure of V6 studied previously by us can be mentioned [51], which shows a ground state degeneracy in the absence of the magnetic field and also two quantum level crossings (taking place at rather high magnetic fields as compared to the case of V12). In V6, the energy gap between the ground state and the first excited state first rises linearly and then decreases linearly with the magnetic field. On the contrary, in V12 the ground state is non-degenerate and the gap is a linearly decreasing function of the magnetic field below $B_{c,1}$. Such a difference causes a totally different magnetocaloric behaviour to emerge for the range of low temperatures and fields (for example the direct MCE for V6 and inverse MCE for V12 studied in the present work), showing the importance of the cluster geometry and magnetic interactions to the thermodynamic properties of clusters.

The present work leaves aside the issues related to the dynamical processes in the studied molecular nanomagnet. They might involve such processes as the energy exchange between the subsystem of localized spins and the underlying lattice [64], leading, for example, to magnetization relaxation phenomenon (particularly important for memory-oriented applications of molecular magnets). Let us note that such effects can be measured, for example, by means of nuclear magnetic resonance methods (see Refs. [23,24,25,26]). From the experimental point of view on magnetocaloric properties, the non-equilibrium physics may affect the results of measurements of MCE quantities and the validity of selected assumptions of equilibrium thermodynamics, for example, the relation between the field derivative of entropy and temperature derivative of magnetization [92]. We believe that our study provides a useful prediction of the equilibrium properties of V12, even though the above-mentioned effects are of importance, for example, for the description of rotational MCE (see Ref. [38]). The inclusion of the lattice component might constitute an interesting task for future developments. On the other hand, the interplay of the spin and lattice subsystems is important rather for the magnetocaloric materials exhibiting first-order magnetic phase transition of the magnetostructural origin [93,94] (which is one of the ways towards maximizing the MCE response).

Other extensions of our present work might involve a study of MCE properties of V12 in the range of higher temperatures (with a model including the remaining V spins that do not contribute to magnetic behaviour below 100 K). In addition, other polyoxovanadates might also constitute interesting subjects of both computational and experimental studies of magnetocaloric properties. Among a variety of experimental works, for the class of cluster polyoxovanadates, a contribution concerning the magnetocaloric properties of V15 molecular magnet [61] is worth mentioning. Another direction of further research might be related to the characterization of the influence of intercluster magnetic couplings on the properties of V12 and other cluster molecular magnets (as shown in Ref. [95]).

## Abbreviations

The following abbreviations are used in this manuscript:

V12	(NH(C${}_{2}$H${}_{5}$)${}_{3}$)${}_{4}$[V${}_{8}^{\mathrm{IV}}$V${}_{4}^{V}$As${}_{8}$O${}_{40}$(H${}_{2}$O)]·H${}_{2}$O
MCE	magnetocaloric effect

## Appendix A. Ground State of the Tetramer for Various Magnetic Fields and the Critical Magnetic Fields

For the investigated tetramer system with anisotropic interactions, the Hamiltonian eigenvalues can be expressed rigorously after exact diagonalization. This procedure allows the analytic discussion of the dependence of the ground-state energy on the applied magnetic field as well as exact calculation of the critical magnetic fields at which the energy levels cross.

For the lowest range of magnetic fields the ground state of the system has total spin S=0. Its energy can be expressed, in general, as a root of the cubic equation (which we do not present here due to its length). However, for the specific case of tetramer with identical interactions isotropic in spin space, $J=J_{1}^{z}=J_{1}^{xy}=J_{2}^{xy}=J_{2}^{z}$, it is reduced to:

(A1) $$E_{0}=2J.$$

In the case of weak anisotropy of spin-space interactions and in the presence of two slightly different couplings (like it happens in V12), the series expansion of the appropriate root of the cubic equation can be performed with the following result (up to the linear terms in anisotropies):

(A2) $$E_{0}≃2J_{1}^{z}\left[1-\frac{1}{3}\left(1-\frac{J_{1}^{xy}}{J_{1}^{z}}\right)-\frac{1}{6}\left(1-\frac{J_{2}^{z}}{J_{1}^{z}}\right)-\frac{1}{3}\left(1-\frac{J_{2}^{xy}}{J_{1}^{z}}\right)\right]=2J_{1}^{z}\left[\frac{1}{6}+\frac{1}{3}\frac{J_{1}^{xy}}{J_{1}^{z}}+\frac{1}{6}\frac{J_{2}^{z}}{J_{1}^{z}}+\frac{1}{3}\frac{J_{2}^{xy}}{J_{1}^{z}}\right].$$

For intermediate magnetic fields, the ground state has S=1 and its energy equals exactly

(A3) $$E_{1}=\frac{1}{2}J_{1}^{xy}+\frac{1}{2}J_{2}^{xy}-g\mu_{B}B.$$

For the highest magnetic fields the magnetic saturation takes place and the spin equals S=2, whereas the energy of the state is exactly equal to:

(A4) $$E_{2}=-\frac{1}{2}J_{1}^{z}-\frac{1}{2}J_{2}^{z}-2g\mu_{B}B.$$

The transition between the state with S=0 and S=1 occurs at the first critical field $B_{c,1}$, which approximately equals:

(A5) $$B_{c,1}≃\frac{|J_{1}^{z}|}{g\mu_{B}}\left(\frac{1}{3}+\frac{1}{6}\frac{J_{1}^{xy}}{J_{1}^{z}}+\frac{1}{3}\frac{J_{2}^{z}}{J_{1}^{z}}+\frac{1}{6}\frac{J_{2}^{xy}}{J_{1}^{z}}\right).$$

For the accepted exchange integrals in V12, the value of this critical field is 13.3 T. For fully isotropic tetramer, with $J=J_{1}^{z}=J_{1}^{xy}=J_{2}^{xy}=J_{2}^{z}$, the first critical field is reduced to $B_{c,1}=\frac{\left|J\right|}{g\mu_{B}}$.

The transition between the state with S=1 and S=2 takes place at the second critical field $B_{c,2}$ having the value of:

(A6) $$B_{c,2}=\frac{|J_{1}^{xy}\left|+\right|J_{1}^{z}\left|+\right|J_{2}^{xy}\left|+\right|J_{2}^{z}|}{2g\mu_{B}}.$$

For the accepted exchange integrals in V12, the value of this critical field is 26.1 T. For fully isotropic tetramer, with $J=J_{1}^{z}=J_{1}^{xy}=J_{2}^{xy}=J_{2}^{z}$, the second critical field is reduced to $B_{c,2}=\frac{2|J|}{g\mu_{B}}$.
